# Recovery of Pituitary and Visual Function After Rathke’s Cleft Cyst Decompression: An 80-case Institutional Experience

**DOI:** 10.1210/jendso/bvaf093

**Published:** 2025-05-20

**Authors:** Haruko Yoshimoto, Masataka Kato, Atsushi Ishida, Hideki Shiramizu, Go Matsuoka, Noriaki Tanabe, Naoko Inoshita, Koji Takano, Masami Ono, Nobuhiro Miki, Masafumi Hamada, Sachiko Tanaka-Mizuno, Shozo Yamada

**Affiliations:** Hypothalamic and Pituitary Center, Moriyama Memorial Hospital, Tokyo 134-0081, Japan; Hypothalamic and Pituitary Center, Moriyama Memorial Hospital, Tokyo 134-0081, Japan; Hypothalamic and Pituitary Center, Moriyama Memorial Hospital, Tokyo 134-0081, Japan; Hypothalamic and Pituitary Center, Moriyama Memorial Hospital, Tokyo 134-0081, Japan; Hypothalamic and Pituitary Center, Moriyama Memorial Hospital, Tokyo 134-0081, Japan; Hypothalamic and Pituitary Center, Moriyama Memorial Hospital, Tokyo 134-0081, Japan; Department of Pathology, Moriyama Memorial Hospital, Tokyo 134-0081, Japan; Department of Endocrinology, Moriyama Memorial Hospital, Tokyo 134-0081, Japan; Department of Endocrinology, Tokyo Clinic, Tokyo 100-0004, Japan; Department of Endocrinology, Tokyo Clinic, Tokyo 100-0004, Japan; Nangyo Inagaki Eye Clinic, Chiba 272-0143, Japan; Laboratory of Epidemiology and Prevention, Kobe Pharmaceutical University, Kobe 658-8558, Japan; Hypothalamic and Pituitary Center, Moriyama Memorial Hospital, Tokyo 134-0081, Japan

**Keywords:** Rathke's cleft cyst, pituitary dysfunction, visual recovery, decompression surgery, AVP deficiency, endoscopic transnasal approach

## Abstract

**Purpose:**

Rathke’s cleft cyst can lead to visual and pituitary dysfunction. Surgical decompression may alleviate these issues, but the decision to operate remains controversial because of the risk of worsening function.

**Methods:**

A retrospective analysis was conducted on 80 patients who underwent endoscopic transnasal transsphenoidal surgery for Rathke’s cleft cyst from April 2018 to April 2024. Clinical outcomes were assessed using magnetic resonance imaging, intraoperative findings, and histopathology to evaluate factors associated with recovery.

**Results:**

After a median follow-up of 13.5 months (interquartile range: 6-30.8 months), anterior pituitary function significantly improved in patients with cysts that did not compress the optic nerve and had minimal thinning of the ganglion cell layer-inner plexiform layer (*P* < .05). Arginine vasopressin deficiency occurred significantly more often in patients with preoperative adult GH deficiency, a history of surgery, and evidence of inflammation in specimens on pathology (*P* < .05). Retinal nerve fiber layer thickness correlated with visual field impairment both before and after surgery, suggesting it may predict visual recovery.

**Conclusion:**

Decompression of Rathke’s cleft cysts can improve anterior pituitary function in patients with smaller cysts and minimal optic nerve pressure. Visual function will likely improve in those with minimal optic nerve fiber layer thinning. Therefore, preoperative optical coherence tomography and visual field testing are mandatory to set expectations regarding potential improvement in vision and anterior pituitary function following surgery. Patients with preoperative adult GH deficiency and a history of surgery are at higher risk of developing postoperative arginine vasopressin deficiency.

Rathke's cleft cyst (RCC) is a nontumoral remnant tissue originating from Rathke's pouch during the embryonic period [1]. It is typically benign and asymptomatic in most cases; however, RCCs can increase in volume due to fluid accumulation or acute hemorrhage into the cyst cavity, leading to compression of the optic nerves and the normal pituitary gland. Consequently, this can decrease visual acuity, visual field defects, and/or pituitary dysfunction. Conversely, spontaneous involution of RCC is occasionally observed [2, 3], and, in some cases [2], the patient's deficits may normalize. However, the resolution of symptoms remains unpredictable [3, 4]. Given the risks associated with surgery, such as the development of new visual or pituitary deficits, the indication for surgical intervention in RCC cases remains controversial [3, 5-7]. In this study, we retrospectively reviewed our surgical cases from a single institution and analyzed various factors influencing postoperative outcomes.

## Patients and Method

This study was approved by the Ethical Committee of Moriyama Memorial Hospital (24007). Among the 95 cases diagnosed with RCC based on preoperative imaging from April 2018 to April 2024, pathology excluded 6 granulomas, 5 craniopharyngiomas, 2 arachnoid cysts, 1 neurocytoma, and 1 unidentified case. A retrospective review was performed on 80 consecutive patients pathologically diagnosed with RCC. Recurrence was defined as regrowth of the cyst, confirmed via magnetic resonance imaging (MRI), which necessitated a change in treatment, such as additional surgery, radiation therapy, or hormone replacement.

### Visual Function

We analyzed visual function using the Humphrey visual field analyzer (ZEISS Humphrey Field Analyzer 3, ver. 1.5.2.431, Carl Zeiss Meditec Inc., Jena, Germany) (RRID: SCR_026000) both preoperatively and postoperatively. Postoperative data were collected from the earliest postdischarge follow-up. Visual field defect (VF) and visual acuity were assessed according to the scales established by El-Mahdy et al [8] (Tables 1 and 2), with improvement or deterioration defined as a change of 1 rank or more. Briefly, rank I indicates normal function, whereas rank V represents no perception of light; visual acuity and visual field deteriorate as the rank increases. After the initial postoperative evaluation, we repeated visual tests whenever the results changed or when patients noticed visual impairment during periodic visits. Visual field mean deviation (VF-MD) and visual field pattern standard deviation (VF-PSD), calculated by the Humphrey Visual Field Analyzer, were recorded as visual field parameters. VF-MD is a measure of retinal sensitivity, with a decrease indicating reduced sensitivity. VF-PSD reflects differences in sensitivity at adjacent test points and correlates well with the degree of visual field loss when the loss is less than 50% [9]. Mean retinal nerve fiber layer (RNFL) thickness and the presence or absence of ganglion cell layer-inner plexiform layer (GCL + IPL) thinning were recorded in each eye using optical coherence tomography (OCT) (ZEISS Cirrus 5000, ver. 11.0.0.29946, Carl Zeiss Meditec Inc.) (RRID: SCR_025999). GCL + IPL thickness parameters were compared to a normal reference range and classified into 3 categories: 5% to 95%, 1% to 5%, and <1% thinning, corresponding to normal-mild, moderate, and severe thinning, respectively. In this study, moderate to severe thinning was determined if any of the six 60° divisions in either eye showed a change.

**Table 1.** Visual acuity assessment (modified from reference 8)

Rank	Visual acuity
I	20/20-20/30
II	20/40-20/80
III	20/100-20/160
IV	20/200-light perception
V	No perception of light

**Table 2.** Visual field assessment (modified from reference 8)

Rank	Visual field
I	Normal
II	Thin scotoma or incomplete quadrantanopia
III	Complete quadrantanopia or incomplete hemianopia
IV	Complete hemianopia with or without additional deficits
V	No perception of light

### Hormonal Examination

The hormonal examination included baseline serum hormone levels, including prolactin (PRL), GH, IGF-1, TSH, free T4, LH, FSH, total testosterone, estradiol (E2), ACTH, and cortisol. These were measured on the day of the operation, on the third or fourth postoperative day, and during outpatient follow-up. Preoperative GH-releasing peptide-2 (GHRP-2) test and TRH-CRH-GnRH tests were performed preoperatively and routinely within the first postoperative week. Hormone concentrations were measured with the Elecsys Prolactine II (Roche Diagnostics, Basel, Switzerland) (RRID: AB_3678553) for PRL, Elecsys hGH (Roche Diagnostics) (RRID: AB_3678554) for GH, Elecsys TSH (Roche Diagnostics) (RRID: AB_3095311) for TSH, Elecsys FT4 IV (Roche Diagnostics) (RRID: AB_3095310) for free T4, Elecsys LH (Roche Diagnostics) (RRID: AB_2920601) for LH, Elecsys FSH (Roche Diagnostics) (RRID: AB_3678555) for FSH, Elecsys ACTH (Roche Diagnostics) (RRID: AB_3678556) for ACTH, and Elecsys Cortisol II (Roche Diagnostics) (RRID: AB_3678557) for cortisol and the instrument used was Cobas Pure e402 (RRID: SCR_026639). The concentration of IGF-1 was measured with Elecsys IGF-1 (Roche Diagnostics) (RRID: AB_3678558) using cobas 8000 <E602> (RRID: SCR_026640). Total testosterone and E2 were measured with ARCHITECT 2nd Generation Testosterone (RRID: AB_2895354, 2P13-28) (Abbott Japan, Tokyo, Japan) and ARCHITECT Estradiol (RRID: SCR_2813911, 7K72-25) (Abbott Japan) using Architect i2000SR (RRID: SCR_026638) (Abbott Japan). The drugs used for the loading tests are GHRP KAKEN 100 Injection (Kaken Pharmaceuticals, Tokyo, Japan) for GHRP-2 tests, TRH Injection (NIPRO ES PHARMA, Osaka, Japan), hCRH Injection (NIPRO ES PHARMA), and LH-RH Injection (NIPRO ES PHARMA) for simultaneous TRH-CRH-GnRH test.

Pituitary dysfunction was diagnosed by low baseline serum hormone levels or a poor response to the tests. The reference range for morning cortisol levels in normal participants was 7.07 to 19.6 μg/dL using the Elecsys Cortisol II assay [10], with levels <9 μg/dL considered indicative of adrenal insufficiency. This is certainly a stringent criterion; however, no discrepancy was found between this baseline value criterion and the results of the postoperative dynamic test. In outpatient follow-up, where the dynamic test is difficult, only cases with a fasting early morning cortisol level of ≥9 μg/dL are considered to have no adrenal hypofunction and are not treated with replacement therapy for clinical safety reasons.

Central hypothyroidism was defined as normal or low TSH and FT4 below the reference level. Central hypogonadism was defined as the presence of LH and FSH levels within the normal reference range or lower, together with low testosterone or E2 levels, incorporating the presence or absence of menstrual cycles in reproductive-age women. In postmenopausal women, central hypogonadism was defined by the absence of the expected physiologic elevation in LH and FSH levels.

A peak GH level ≤9 ng/mL in response to the GHRP-2 test was used to diagnose severe adult GH deficiency (AGHD) [11]. Although peak serum GH is significantly influenced by both age and body mass index (BMI), as reported by Yuen et al [12], the GHRP-2 test has been shown to classify individuals with severe AGHD and healthy participants diagnosed by the ITT without overlap when the cutoff value for peak GH concentration of 9 μg/L (calibrated with the recombinant World Health Organization 98/574 standard) is used [11]. In the study validating the accuracy of the GHRP-2 test, the maximum BMI of participants in the normal control group was 36.2 kg/m^2^, whereas that of the severe GHD group was 30.3 kg/m^2^. Among the patients we tested with the GHRP-2 test, only 1 had a BMI over 30 kg/m^2^, and this patient was diagnosed as not having severe AGHD based on the GHRP-2 test results. Based on this evidence, we believe BMI does not influence the diagnosis of AGHD or the results of this paper's analysis.

We judged hormonal remission by normalization of baseline values and completion of drug administration.

Arginine vasopressin (AVP) deficiency was diagnosed clinically based on large volumes of dilute urine, which could be promptly controlled by oral desmopressin in the outpatient setting. A 3% salt water solution or water restriction test was performed if indicated. AVP deficiency was confirmed during the perioperative period if urine output exceeded 1.5 L within 6 hours, with a specific gravity of less than 1.005, despite water restriction.

### Neuroimaging Examination

All patients underwent MRI using a 3.0T scanner (Ingenia Elition, Philips Healthcare) (RRID: SCR_025998) with 2.0-mm slice thickness before surgery and within 1 week postoperatively. T1-weighted imaging was performed at repetition time 491 ms, and T2-weighted imaging at repetition time 3000 ms and echo time 80 ms.

The cyst contents were classified based on their intensity on T1-weighted imaging and T2-weighted imaging as isointense, hypointense, hyperintense, or mixed (including Niveau formation) relative to the pituitary gland or cerebral gray matter if the pituitary gland was very thin. The craniocaudal height of the cyst from the sellar floor was measured, and preoperative chiasmal deformity due to cyst compression was also evaluated.

### Operative Technique

Sixty-three patients underwent cyst decompression via cyst puncture using standard endonasal transsphenoidal surgery with endoscopy. To minimize invasiveness to the nasal cavity, a single-nostril approach was employed in principle. After dural opening, cyst decompression was performed by making a small incision in the cyst wall, allowing spontaneous outflow of the cyst contents. Gross findings of the cyst contents were classified as pus-like, xanthochromic-serous, transparent-mucous, or cerebrospinal fluid (CSF)-like.

A cyst wall specimen was taken using a small Rongeur while avoiding traction on the pituitary gland. If the pathological diagnosis of RCC was probable, no further cyst wall resection was performed. The perforated hole was left open in cases where cerebrospinal fluid did not emerge after drainage of the cyst contents despite the Valsalva maneuver (Group M). After the 24th case, we introduced an adjunctive technique known as the “mucosa coupling method” [13] to prevent the recurrence of fluid accumulation (Group MC). In cases of CSF leakage, the dural opening was closed with abdominal fat, and the sellar floor was reconstructed using bone, Neoveil, and fibrin glue, with or without sphenoid sinus mucosal layer coverage (Group MF). After the 29th case, we inspected the cyst cavity using the “diving method” [14].

Seventeen patients underwent extended endonasal transsphenoidal surgery (Ex TSS) because the cyst was large, requiring suprasellar inspection for the preservation of normal tissue or more extensive cyst wall removal for pathological diagnosis or to prevent recurrence (Group Ex).

We did not prescribe high-dose glucocorticoids perioperatively as therapy.

### Headache

We considered headaches to be the indication for surgery only when patients' symptoms did not improve despite meticulous treatments and when they had trouble going to school or working. Postoperative improvement was judged when patients no longer needed painkillers and could lead their daily lives as usual.

### Pathological Examination

Operative specimens were fixed in 10% buffered formaldehyde, dehydrated in graded ethanol, embedded in paraffin, and examined using routine histological methods. The pathological diagnosis of RCC was confirmed by identifying ciliary columnar epithelium. Atypical findings such as squamous metaplasia or inflammatory cell infiltration were also noted.

### Statistical Analysis

Fisher's exact test was used to analyze correlations between categorical variables, whereas Welch's *t*-test was used to compare the means of 2 groups. Data were collected using HAD software [15]. A *P* value of <.05 was considered statistically significant.

## Results

Eighty patients were eligible for this study, including 62 females (77.5%). The age range of the patients was 16 to 84 years, with a median (interquartile range) of 57.5 (50.8-70.8) years. The follow-up period ranged from 0.5 to 66 months, with a median (interquartile range) of 13.5 (6-30.8) months. The indications for surgery were as follows: impaired visual function (43.5%), intractable forehead headache (17.5%), anterior pituitary dysfunction (16.3%), RNFL thinning detected by OCT (12.5%), need for pathological diagnosis (6.3%), and cyst enlargement during follow-up (1.3%). Patients with a single headache episode, even with the characteristic features of RCC on MRI, were typically not operated on, as such cases can be self-limiting and may regress spontaneously [4]. Twelve patients (15.0%) underwent surgery because of symptomatic relapse after a previous operation. Patients in Group Ex had a significantly higher rate of prior surgery than those in other groups (Group M 0%, Group MC 9.1%, Group MF 6.7%, Group Ex 47.1%; *P* = .000).

Postoperative MRI confirmed decompression of RCC in all patients, and permanent pathological specimens confirmed the diagnosis of RCC. Concomitant squamous metaplasia was observed in 11 patients (13.8%), and inflammatory changes were present in 23 patients (28.8%). These characteristics are summarized in Table 3.

**Table 3.** Patients' characteristics

Number of patients	80
Female	62 (77.5%)
Age (y), median (interquartile)	16-84, 57.5 (50.8-70.8)
Follow-up period (months), median (interquartile)	0.5-66, 13.5 (6-30.8)
Operative indication	
Impaired visual function	37 (46.3%)
Intractable headache	14 (17.5%)
Anterior lobe dysfunction	13 (16.3%)
Retinal nerve fiber layer thinning	10 (12.5%)
Need for pathological diagnosis	5 (6.3%)
Suprasellar extension during follow-up	1 (1.3%)
Preoperative AVP deficiency (not operative indication alone)	12 (15.0%)
Preoperative anterior pituitary deficit	
Adrenal insufficiency	22/78 (28.2)
Thyroidal insufficiency	29/79 (36.7)
Gonadal insufficiency	18/69 (26.1)
Severe adult growth hormone deficiency	33/70 (47.1)
Hyperprolactinemia	22/79 (27.8)
History of previous operation	12 (15.0%)
Radiological characteristics	
T1WI low	15 (18.8%)
Iso	20 (25.0%)
High	34 (42.5%)
Mixed	11 (13.8%)
T2WI low	10 (12.5%)
Iso	10 (12.5%)
High	42 (52.5%)
Mixed	18 (22.5%)
Height of the cyst (mm)	16.1 ± 5.4
Chiasmal deformity due to cyst compression	65 (81.3%)
Operative technique	
Simple membranectomy	15 (18.8%)
Simple membranectomy and fat graft closure	15 (18.8%)
Simple membranectomy with mucosa coupling method	33 (41.3%)
Any simple membranectomy w/steroid water irrigation	22/63 (34.9%)
w/observation of intracavity with the diving method	18/63 (28.6%)
Ex TSS with cyst wall resection	17 (21.3%)
Gross finding of cyst content	
Pus-like	34 (42.5%)
Transparent-mucous	34 (42.5%)
Xanthochromic-serous	6 (7.5%)
CSF-like	4 (5.0%)
Lost before confirmation	2 (2.5%)
Postoperative CSF leakage	2 (2.5%)
Postoperative hemorrhage	1 (1.3%)
Pathological findings	
Inflammation	23 (28.8%)
Squamous metaplasia	11 (13.8%)
Recurrence (regrowth after this operation requiring any change in treatment)	5 (6.3%)

Abbreviations: CSF, cerebrospinal fluid; Ex TSS, extended endonasal transsphenoidal surgery; T1WI, T1-weighted magnetic resonance imaging; T2WI, T1-weighted magnetic resonance imaging.

### Pre- and Postoperative Evaluation

#### Anterior pituitary hormones

Of the 80 cases, 77 (96.3%), 78 (97.5%), 64 (80.0%), 68 (85.0%), and 78 (97.5%) patients were able to undergo preoperative and postoperative endocrinological assessments in the ACTH-cortisol axis, TSH-thyroid hormone axis, GH-IGF-1 axis, LH/FSH-sex steroid axis, and PRL, respectively.

**Figure 1.**
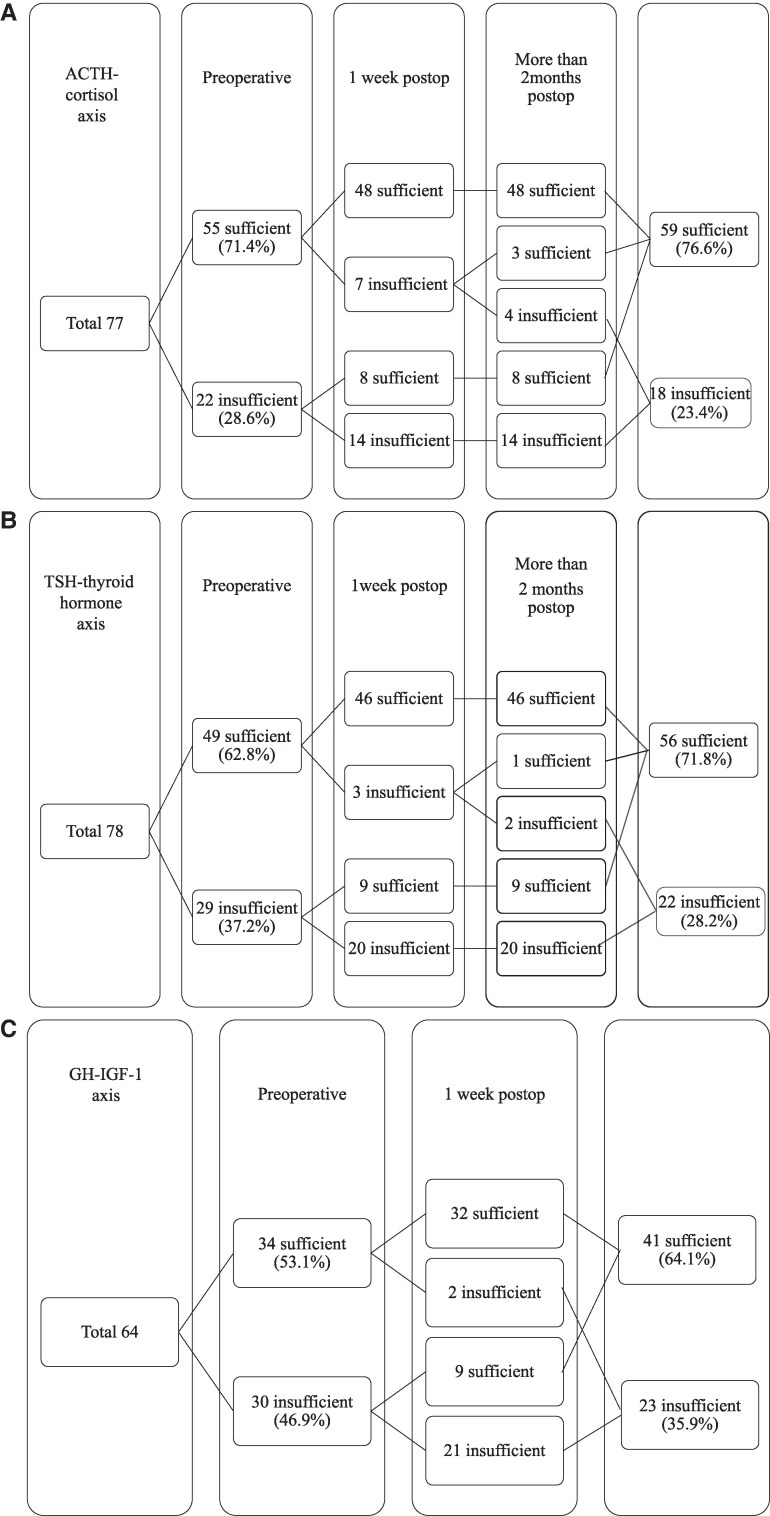

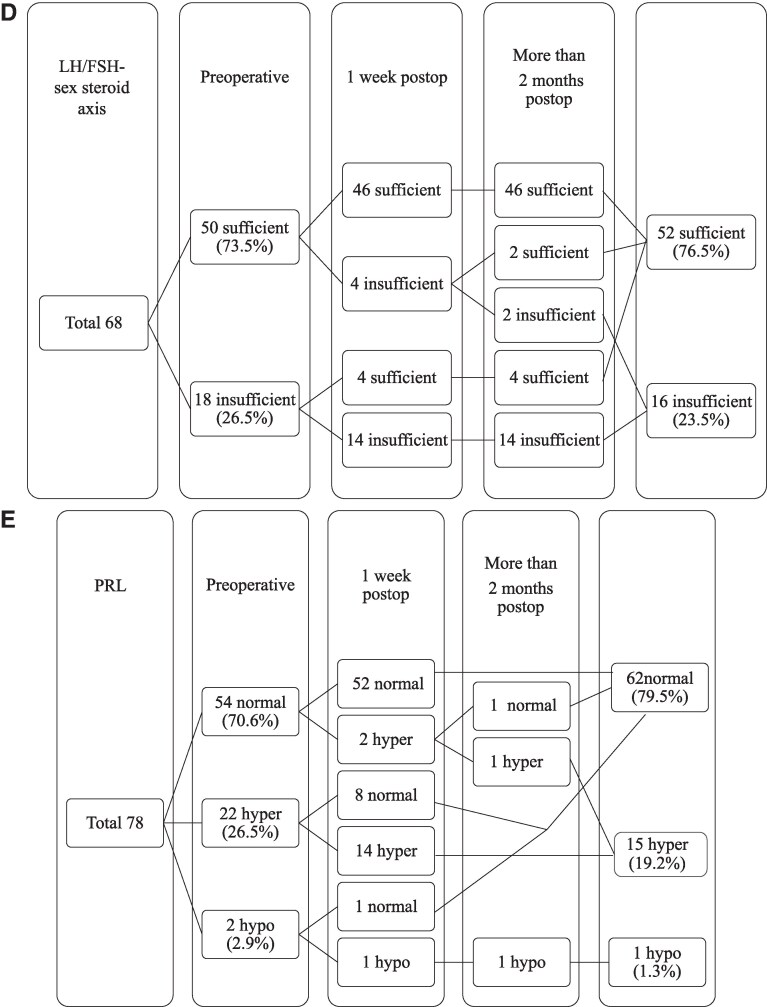
Postoperative improvement and deterioration of the ACTH-cortisol axis (A), TSH-thyroid hormone axis (B), GH-IGF-1 axis (C), LH/FSH-sex steroid axis (D), and PRL (E). Abbreviations: ACTH = adrenocorticotropic hormone; TSH = thyroid stimulating hormone; GH = growth hormone; IGF-1= insulin-like growth factor; LH = luteinizing hormone; FSH = follicle stimulating hormone; PRL = prolactin.

The proportion of ACTH-deficient cases was 28.6% preoperatively, decreasing to 23.4% postoperatively (Fig. 1A). TSH deficiency was present in 37.2% of cases before surgery and decreased to 28.2% after surgery (Fig. 1B). GH deficiency was present in 46.9% of cases preoperatively and decreased to 35.9% postoperatively (Fig. 1C). LH/FSH deficiency was present in 26.5% of cases preoperatively, decreasing to 23.5% postoperatively (Fig. 1D). Hypersecretion of PRL was observed in 26.5% of cases before surgery, decreasing to 19.2% postoperatively (Fig. 1E).

Of the 80 patients, endocrinological evaluations were completed in 64 patients. Overall, 21 of 40 patients (52.5%) with any anterior pituitary dysfunction, excluding preoperative elevated or reduced PRL, showed improvement and no longer required hormone replacement therapy.

Among 22 cases with normal preoperative anterior pituitary function confirmed through a full endocrinological study, 7 patients developed new hormonal insufficiency postoperatively. Five of these cases were transient, resolving within a mean of 11.6 ± 11.1 months (range: 3-30 months), leaving 2 cases of persistent postoperative hormone deficiency. In cases with preoperative anterior pituitary deficiency, 4 patients developed additional hormone deficiencies postoperatively. In total, 6 patients (6/80 = 7.5%) developed persistent new or additional postoperative anterior pituitary deficiencies, with 5 of these patients (excluding 1 with GH deficiency) requiring hormone replacement therapy.

### Factors Related to Postoperative Hormone Prognosis

The average age of patients who achieved remission was significantly younger than those who did not (mean age, 51.1 ± 16.6 years vs 67.2 ± 12.0 years, respectively; *P* = .001). The remission rate was also significantly higher in the group without preoperative chiasmal deformity owing to cyst compression than in the group with deformity (7/14 = 50.0% vs 14/63 = 22.2%, respectively; *P* = .048).

Among the 39 patients who underwent OCT, the remission rate was significantly higher in those with no thinning of the GCL + IPL on either side compared to that of those with thinning (5/9 = 55.6% vs 5/30 = 16.7%, respectively; *P* = .032). Similarly, remission was higher in patients with no or moderate GCL + IPL thinning than in those with severe thinning (8/15 = 53.3% vs 2/24 = 8.3%, respectively; *P* = .003) (Table 4).

**Table 4.** Remission of anterior lobe dysfunction except for PRL

	Present (%)	Not (%)	*P*
Chiasmal deformity due to cyst compression	14/63 (22.2)	7/14 (50.0)	.048
Moderate or severe thinning of GCL + IPL (n = 39)	5/30 (16.7)	5/9 (55.6)	.032
Severe thinning of GCL + IPL (n = 39)	2/24 (8.3)	8/15 (53.3)	.003

Abbreviations: GCL + IPL, ganglion cell layer-inner plexiform layer; PRL, prolactin.

New persistent anterior pituitary deficits developed in 7.5% of patients (6/80). No significant correlation was found between the development of new anterior pituitary deficits and factors such as patient age, preoperative AVP deficiency, anterior pituitary dysfunction, hyperprolactinemia, cyst height or intensity on MRI, preoperative chiasmal deformity, or GCL + IPL thinning.

Regarding the surgical technique, Group Ex had a significantly higher incidence of new postoperative anterior pituitary deficits than Group M and Group MC combined, who underwent simple membranectomy without CSF leakage (23.5% vs 2.1%, *P* = .015). Additionally, a history of operations was significantly associated with new postoperative anterior pituitary dysfunction (2 of 68 cases of initial surgery [2.9%] vs 4 of 12 cases with a history of surgery [33.3%]; *P* = .004). Patients in Group Ex had a significantly higher rate of previous surgery than those in Group M and Group MC combined (12.3% vs 4.6%; *P* = .001).

AVP deficiency was present preoperatively in 12 cases, but none of these cases recovered after cyst decompression. Sixteen cases (23.5%) developed new-onset postoperative AVP deficiency. Among these, 9 cases (60.0%) experienced remission. The time from onset of AVP deficiency to remission ranged from 0.25 to 12 months, with a mean duration of 4.1 ± 4.5 months.

Postoperative AVP deficiency occurred significantly more often in patients with preoperative AGHD (39.1% vs 11.1%; *P* = .024), those with a history of surgery (50.0% vs 17.2%; *P* = .033), and finding of inflammation in the specimen on pathology (41.2% vs 15.7%; *P* = .043) (Table 5). When categorized by cyst content, AVP deficiency occurred in 44.0% of cases with pus-like mucous contents, compared to significantly fewer cases with transparent-mucous contents (9.4%) (Table 6). Only 1 of the 15 patients with T1 low-intensity cysts developed transient AVP deficiency postoperatively. The incidence of new AVP deficiency in patients with T1 low-intensity cysts was not statistically significant compared to that in non-T1 low-intensity groups (6.7% vs 26.42%; *P* = .161). No association was found between postoperative AVP deficiency and patient age, cyst height, surgical technique, or the presence of preoperative visual dysfunction.

**Table 5.** Frequency of postoperative transient or persistent AVP deficiency

	Present	Not	*P*
Preoperative AGHD	9/24 (69.2)	4/36 (30.8)	.024
Previous operation	5/10 (50.0)	10/59 (17.0)	.033
Inflammation in specimen on pathology	7/17 (41.2)	8/51 (15.7)	.043

Abbreviations: AGHD, adult GH deficiency; AVP, arginine vasopressin.

**Table 6.** Frequency of postoperative transient or persistent AVP deficiency and cyst content

	AVP deficiency (%)	*P*
Pus-like	11/25 (44.0)	—
Transparent-mucous	3/32 (9.4)	.004^*a*^
Xanthochromic-serous	0/6 (0)	.066
CSF-like	1/4 (25.0)	.622

Abbreviations: AVP, arginine vasopressin; CSF, cerebrospinal fluid.

^a^Significantly infrequent compared to those with pus-like cyst content.

In cases with an observation period of at least 12 months, transient AVP deficiency was significantly more common in older patients (transient AVP deficiency: 71.1 ± 6.4 years vs persistent AVP deficiency: 48.4 ± 18.1 years; *P* = .046). Additionally, persistent AVP deficiency was significantly more common in patients without preoperative AGHD (3/3 in non-AGHD patients [100%] vs 2/9 in AGHD patients [22.2%]; *P* = .045).

### Visual Function

Preoperatively, 37 patients had impaired visual function because of cyst compression of the optic nerve or chiasma. Static visual field testing was performed before and after surgery on 63 eyes in 32 patients, and visual acuity testing was conducted on 51 eyes in 26 patients, including 3 patients without preoperative visual impairment.

Among the 39 eyes with preoperative visual field defects, 89.7% (35/39) showed an improvement of at least 1 rank, 7.7% (3/39) exhibited no change, and 2.5% (1/39) worsened by at least 1 rank at the first postoperative examination (Table 7). Among the 10 eyes with preoperative impaired visual acuity, 90.0% (9/10) improved by at least 1 rank, whereas 10.0% (1/10) showed no change, and no eyes worsened (Table 8). In 50 eyes, including those without obvious preoperative visual field defects, postoperative VF-MD values significantly improved compared to preoperative values (−4.22 ± 5.66 vs −1.39 ± 4.47; *P* = .000), whereas VF-PFD values significantly decreased (5.20 ± 4.64 vs −2.16 ± 1.85; *P* = .000).

**Table 7.** Pre- and postoperative visual field rating (number of patients)

	Postoperative visual field rank			
	I	II	III	IV
Preoperative visual field rank				
I	24	0	0	0
II	11	2	1	0
III	17	2	1	0
IV	0	4	1	0

**Table 8.** Pre- and postoperative visual acuity rating (number of patients)

	Postoperative visual acuity rank			
	I	II	III	IV
Preoperative visual acuity rank				
I	41	0	0	0
II	6	1	0	0
III	2	0	0	0
IV	1	0	0	0

**Figure 2.**
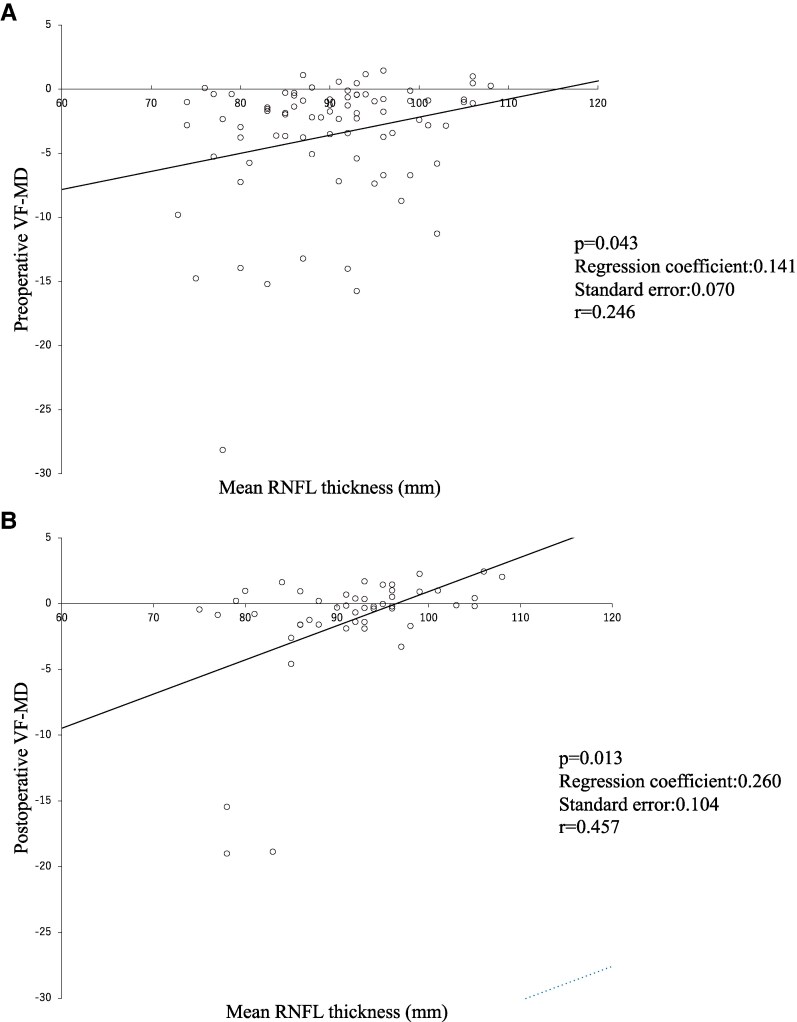

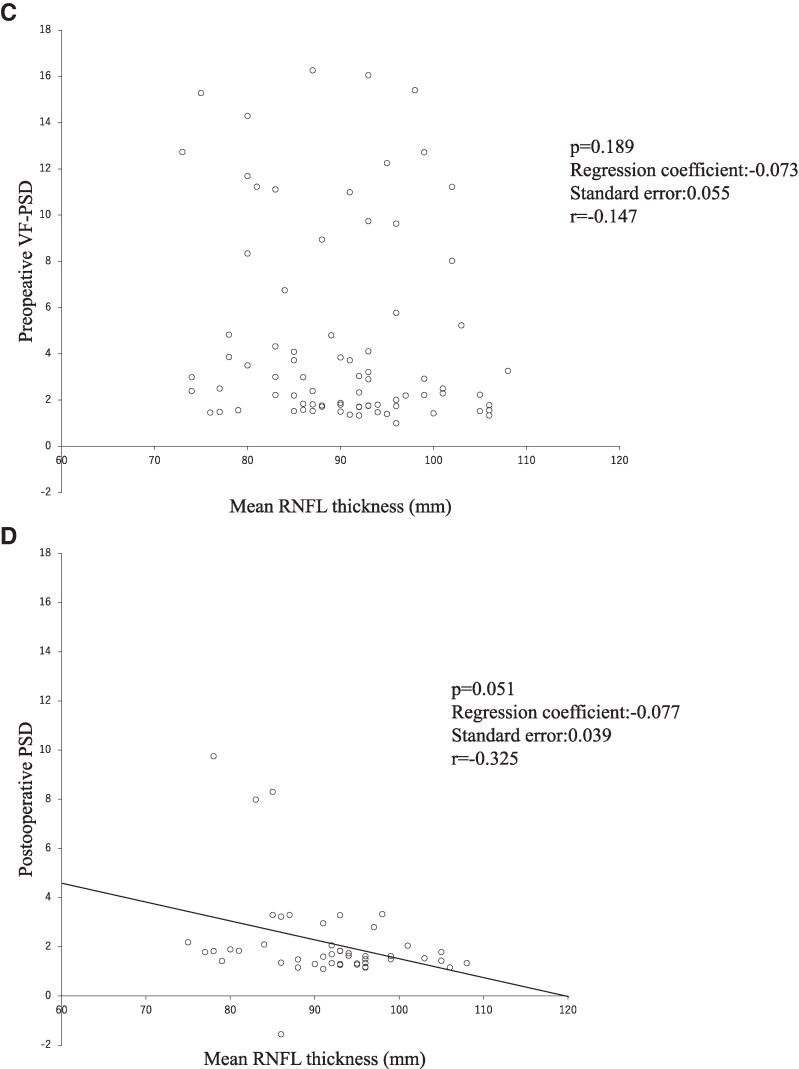
Mean RNFL values correlated with both pre- and postoperative VF-MD values (A, *P* = .043; B, *P* = .013, respectively) and the correlation coefficient increased postoperatively (*r* = 0.246 vs 0.457). VF-PFD did not correlate preoperatively (C, *P* = .189, *r* = 0.147), but there was a trend toward correlation postoperatively (D, *P* = .051, *r* = 0.325). These indicate that surgical decompression can improve visual function to a level commensurate with RNFL. Abbreviations: RNFL, retinal nerve fiber layer thickness; VF-MD, visual field mean deviation; VF-PFD, visual field pattern standard deviation.

Factors related to postoperative visual function improvement were analyzed in 49 eyes for which preoperative OCT was performed. Mean RNFL values were significantly correlated with preoperative and postoperative VF-MD values (Fig. 2A, *P* = .043; Fig. 2B, *P* = .013), with a stronger postoperatively correlation (*r* = 0.246 vs 0.457). VF-PFD did not show a significant preoperative correlation (Fig. 2C; *P* = .189, *r* = 0.147), but there was a trend toward correlation postoperatively (Fig. 2D; *P* = .051, *r* = 0.325). These results suggest that surgical decompression can improve visual function in proportion to RNFL recovery.

### Headache

Thirty patients reported headaches preoperatively, 23 of whom cited headaches as their primary complaint. Patients with headaches were significantly younger than those without (45.1 ± 15.9 vs 60.8 ± 16.6 years; *P* = .000), and the cyst height in patients with headaches was significantly smaller compared to that of those without headaches (12.9 ± 4.8 mm vs 17.9 ± 4.8 mm; *P* = .000). Multiple regression analysis revealed that age and cyst height independently correlated with the presence of headaches (age: *P* = .017; cyst height: *P* = .004).

Postoperatively, headache symptoms improved markedly in 22 of 30 patients (73.3%). Patients who experienced headache remission were significantly younger than those without remission (41.0 ± 15.3 vs 56.3 ± 12.0 years, respectively; *P* = .012). No significant differences were found in cyst height, inflammatory findings on pathology, or the surgical technique between patients whose headaches improved and those whose headaches did not.

### Recurrence

Five recurrences (6.3%) required additional treatment because of cyst regrowth. Three patients (3.8%) experienced visual deterioration from cyst enlargement and underwent cyst wall removal via extended transsphenoidal surgery (Ex TSS). One patient (1.3%) received CyberKnife treatment, and 1 patient (1.3%) developed panhypopituitarism in addition to preexisting AVP deficiency due to transient cyst enlargement. Patients with recurrence had significantly larger cysts than those without recurrence (19.7 ± 2.5 mm vs 15.6 ± 5.3 mm; *P* = .015). However, no significant differences were observed in age, sex, surgical procedure, preoperative AVP deficiency, anterior pituitary hypofunction, or histopathological findings (eg, squamous metaplasia, inflammation) between patients with and without recurrence.

### Intraoperative CSF Leakage

Factors associated with intraoperative CSF leakage were examined in cases excluding Group Ex. Cases with intraoperative CSF leakage had significantly larger cysts than those without leakage (18.8 ± 4.0 mm vs 14.9 ± 5.8 mm, respectively). The incidence of intraoperative CSF leakage was significantly lower in cases without chiasmal deformity compared to that of those with deformity (0/15 [0%] vs 15/48 [31.3%]; *P* = .013). Intraoperative CSF leakage occurred significantly more frequently in cases where a diving technique was used for intracavity observation (8/17 [47.1%] vs 7/46 [15.2%]; *P* = .017) (Table 9).

**Table 9.** Percentage of intraoperative CSF leakage excluding Group Ex

	Yes (%)	No (%)	*P*
Chiasmal deformity due to cyst compression	15/48 (31.3)	0/15 (0)	.013
Diving technique	8/17 (47.1)	7/46 (15.2)	.017

Abbreviations: CSF, cerebrospinal fluid; Group Ex, patient group adopted extended endonasal transsphenoidal surgery.

### Other Operative Complications

Operative complications, aside from pituitary function deterioration, included 1 case of hematoma and 2 cases of postoperative CSF leakage. The small postoperative hematoma around the optic nerve resolved within 8 days after surgery, with the patient's transient visual deterioration also improving. Both cases of postoperative CSF leakage had preexisting intraoperative CSF leakage and were successfully reconstructed during surgery without any lasting complications.

## Discussion

This study revealed several key findings: anterior pituitary dysfunction (excluding PRL) improved by more than one axis in 52.5% of cases, whereas 7.5% experienced additional postoperative anterior pituitary deficits. Among patients with preoperative visual field defects, 89.7% showed at least a 1-rank improvement postoperatively. No patients with preoperative AVP deficiency recovered after cyst decompression. However, 18.8% of patients developed new postoperative AVP deficiency, and 60% of these cases were remitted within 12 months. Cyst recurrences requiring further treatment occurred in 6.3% of patients.

### Anterior Pituitary Function and AVP Deficiency

Although visual dysfunction is widely accepted as an indication for surgery in RCC cases [16], the role of cyst decompression in recovering pituitary function remains controversial. Castle-Kirszbaum et al conducted a systematic review of a series involving 20 or more cases and reported an overall improvement in anterior pituitary function (excluding PRL) in 30.2% (range: 0-68.8) of cases, with 6.9% (range: 0-19.2%) developing new anterior pituitary dysfunction postoperatively [6].

Previous discussions of postoperative pituitary deficiency have largely focused on differences in surgical techniques [5, 17, 18]. However, findings remain inconsistent as to whether gross total cyst removal effectively reduces recurrence rates. Several studies have suggested that factors such as larger cyst size [5, 19], suprasellar extension [5], intrasellar cysts, and gelatinous cyst contents [20] are linked to preoperative anterior pituitary dysfunction [5, 19, 20] or postoperative deterioration of pituitary function [5]. Additionally, the female gender has been reported as an ameliorating factor [21].

In this study, preoperative anterior pituitary dysfunction was more likely to improve in cases where the cyst was small (without chiasmal deformity), the compression was not prolonged (no significant thinning of the GCL + IPL), and in younger patients.

Postoperative anterior lobe dysfunction was significantly more common in patients with a history of previous surgery. Furthermore, Group Ex demonstrated a higher rate of new anterior pituitary dysfunction postoperatively compared to Groups M and MC combined. However, these findings did not reach statistical significance in multivariate analysis.

Regarding new postoperative AVP deficiency, Castleman et al reported in their systematic review that transient AVP deficiency ranged from 0% to 35.6%, with an overall incidence of 13.9%, whereas permanent AVP deficiency ranged from 0% to 24.2%, with an overall incidence of 4.8%. Predictors of new postoperative AVP deficiency reported in the literature include suprasellar extension [5, 22] and gross total resection of the cyst [17]. In this study, risk factors for new postoperative AVP deficiency were preoperative AGHD, inflammatory findings on pathological examination, and a history of previous operations. These findings suggest a mechanism where the posterior lobe and vasopressin neurons may have been impaired and vulnerable before surgery. Following decompression, an acute extension of the pituitary stalk might occur, inducing traction damage on the axons of vasopressin neurons at a proximal site (leading to Wallerian degeneration). The finding that postoperative AVP deficiency is more likely to remit in patients with preoperative AGHD and older individuals appears paradoxical. Maghnie et al studied 15 043 children with GH deficiency, of whom 2361 cases showed anatomical abnormalities, including 25 cases of RCC on MRI. Among these 2361 cases, 459 had ectopic posterior pituitary formation alongside hypoplasia of the anterior lobe and stalk [23]. In our series, older patients or those with deteriorating anterior pituitary function (AGHD) because of RCC may have developed compensatory mechanisms, such as ectopic posterior pituitary formation, allowing for earlier recovery from AVP deficiency.

### Visual Function

In the systematic review by Castle-Kirszbaum et al, 76.4% of patients (40.9%-100%) showed improved visual function following decompression for visual impairment caused by RCC [6]. This high rate of improvement supports the indication of surgery for RCC when visual function is impaired [16]. However, the extent of visual improvement expected from decompression surgery has not been widely described in the literature. In cases of compressive optic nerve disease, preoperative RNFL thickness has been shown to predict postoperative visual outcomes [24]. This study demonstrated that surgical decompression in RCC can lead to visual improvement corresponding to the RNFL thickness, similar to other optic nerve compressive diseases.

Timely surgery can enhance patients' quality of life by improving visual function.

Kinoshita et al reported the natural history of 229 nonsurgical RCC cases with a median observation period of 36.6 months. Their study found that asymptomatic patients or those whose primary complaint was acute headache rarely experienced deterioration, even after long-term follow-up. Considering the frequency of endocrinological deterioration after surgery, they concluded that it is reasonable to follow patients for up to 5 years if they are asymptomatic and for several months if acute headache is the only symptom.

We agree with their conclusions, especially in light of surgical risks and that some RCC cases show symptomatic relief owing to spontaneous regression [2, 4]. However, their report had unclear inclusion criteria for nonsurgical follow-up, and they included fewer symptomatic cases compared to the present study.

Headache, pituitary hypofunction [16], and visual disturbances such as noncomplementary binocular visual field deficits or reduced visual field sensitivity in the better eye are known to lower patients' quality of life [16, 25]. Timely cyst decompression can alleviate these symptoms in selected patients.

Indeed, the indication for surgical treatment of RCC without optic nerve dysfunction is still controversial. We have empirically shown that anterior pituitary function improves in the early stages of the disease and have performed cyst-release surgery mainly in patients with a clear anterior lobe dysfunction onset. As a result, this study showed that remission of at least 1 axis of the anterior pituitary function was observed in 52% of the patients.

However, there are certainly cases in which anterior pituitary function does not improve, and our analysis suggests that those with thinning of the G CL + IPL are considered to have had long-term invasion of the pituitary gland, making surgery to improve anterior lobe function less significant. This is also the first time we have reported this. We also showed that postoperative AVP deficiency is a possible complication in patients with preoperative AGHD and in patients who underwent reoperation.

This study found that preoperative patient-specific factors significantly influence postoperative pituitary function. For instance, in initial cases of RCC without preoperative AGHD, where patients presented with reduced visual function, cyst decompression has a high likelihood of improving visual function without causing pituitary dysfunction. In contrast, younger, recurrent cases with AGHD, impaired visual function, and thinning of the GCL + IPL and RNFL may experience persistent AVP deficiency and new anterior pituitary dysfunction postoperatively without satisfactory restoration of visual function.

RCC is a biologically heterogeneous condition that can sometimes be complicated by inflammation and/or squamous metaplasia [16, 26], and postoperative recurrence is not uncommon. Although cyst opening may prevent further pituitary dysfunction caused by cyst inflammation [26], a thorough risk assessment based on clinical presentation and imaging findings is essential in determining the surgical indication, considering the patients' quality of life.

Recurrence is often debated in the context of surgical technique. Some studies suggest that total cyst wall removal is less likely to result in recurrence [19, 20, 27, 28], whereas others argue that recurrence rates are similar between radical and subtotal removal but with increased complications in radical removal [5, 17, 18]. Factors such as the use of fat and/or fascial graft for closure [18, 29], the presence of squamous metaplasia in the cyst wall [21, 28-31], suprasellar extension [22], and postoperative residual cyst [7, 31] have been reported as risks of recurrence.

The median follow-up in this study was 13.5 ± 17.9 months, which is shorter than the recommended duration for assessing recurrence [30, 32], making it difficult to draw firm conclusions about recurrence in this study.

We observed only 2 cases of postoperative CSF leakage. Although the rate of intraoperative CSF leakage was higher in patients undergoing the diving technique, this method may have helped avoid postoperative CSF leakage in some cases. However, further investigation is needed to clarify this association.

Castle-Kirszbaum et al reported that postoperative olfactory dysfunction reduces quality of life [6]. However, in principle, we avoid this complication by using only 1 nostril for the surgical approach.

## Conclusion

Although RCC is not life-threatening in most cases, deterioration of pituitary or visual function significantly impacts patients' quality of life. Cyst decompression can improve anterior pituitary function, particularly in patients with smaller cysts and a shorter duration of optic nerve compression. Visual function is also more likely to improve in cases with less severe optic nerve fiber layer thinning. Preoperative OCT is mandatory, along with visual field testing, to set expectations for vision and anterior pituitary function improvement following surgery. The surgical indication depends on the patient's condition. However, patients with preoperative AGHD and those with a history of previous surgeries are at higher risk of developing postoperative AVP deficiency, which may persist, particularly in younger patients or those in their early 60s.

### Limitations

The absence of comprehensive endocrinological and ophthalmological evaluations in all patients may have limited the statistical power of this study. Additionally, this study has a relatively short postoperative follow-up period. However, we believe that our study is groundbreaking because it is the first paper to show that improvement in postoperative visual and anterior lobe function in RCC can be predicted from preoperative OCT findings. Indeed, several surgical techniques were applied, allowing us to examine whether deterioration or improvement in pituitary function and improvement in optic nerve function depended on the surgical technique. This highlights the need for larger studies with longer follow-up durations to provide a more accurate analysis of long-term outcomes.

## Data Availability

Some or all of the datasets generated and analyzed during this study are not publicly available but are available from the corresponding author on reasonable request.

## Abbreviations

AGHD: adult GH deficiency
AVP: arginine vasopressin
BMI: body mass index
CSF: cerebrospinal fluid
E2: estradiol
GCL: ganglion cell layer
GHRP-2: GH-releasing peptide-2
IPL: inner plexiform layer
MRI: magnetic resonance imaging
OCT: optical coherence tomography
PRL: prolactin
RNFL: retinal nerve fiber layer
VF: visual field
VF-MD: visual field mean deviation
VF-PSD: visual field pattern standard deviation
